# Knowledge and Practice on Malaria among Korean Soldiers in Nearby Demilitarized Zone in South Korea

**Published:** 2017-09

**Authors:** Yeong Seon HONG, Dong-Kyu LEE, Jin-Han YOON, Aeree SOHN

**Affiliations:** 1. Health and Welfare Graduate School, Sahmyook University, Nowon-Ku, Seoul, Republic of Korea; 2. Dept. of Biological Sciences, School of Health & Environment, Kosin University, Wachi-ro Yeongdo-gu Busan, Republic of Korea; 3. Korea Health Project Evaluation Research Institute, Sahmyook University, 26-21 Kongneung-Dong, Nowon-Ku, Seoul, Republic of Korea; 4. Dept. of Health Management, Sahmyook University, 26-21 Kongneung-Dong, Nowon-Ku, Seoul, Republic of Korea

**Keywords:** Malaria, DMZ, Knowledge, Attitude, Practice

## Abstract

**Background:**

The aim of this study was to provide evidence for developing intervention and effective management for the prevention of malaria based on epidemiological analysis and assessment of Korean soldiers’ knowledge on malaria and malaria preventive behavior.

**Methods:**

The data were collected from 294 Korean soldiers nearby the demilitarized zone in Gyeonggi Province in 2016. Multiple regression analyses were applied for statistical analysis.

**Results:**

The level of education (β=.24, *P*<.001), educational experience on malaria (β=.21, *P*<.001) and ranks like corporal (β=.13, *P*<.05), and sergeant (β=.13, *P*<.05) were observed to be associated with the level of knowledge of malaria (F=9.62, *R*^2^=.12, *P*<.001). Knowledge of malaria (β=.25, *P*<.001) and malaria education experience (β=.22, *P<*.001) were the factors that influenced malaria prevention behavior and practice (F=12.45, *R*^2^=.18, *P*<.001).

**Conclusion:**

The level of knowledge and education experience was associated with malaria prevention practice. Therefore, malaria education is very important for the soldiers in nearby DMZ for prevention of malaria. The findings provide implications for the development of intervention programs focusing on increasing the levels of knowledge and practices related to malaria.

## Introduction

Malaria is one of the top six influential tropical diseases according to WHO. Each year more than 3 billion people are at risk of being infected with malaria, 2 million cases of malaria have occurred globally, and 627000 deaths have been reported to occur each year (1). Malarial infection can cause growth retardation, low birth weight, anemia, or infant death. Women with malaria in pregnancy are at a higher risk for development of acute anemia, premature delivery, or miscarriage, and malaria in HIV/AIDS patients accelerates HIV disease progression. Recently, malaria control and treatment have emerged as imperative issues because re-emergence of malaria in temperate regions in developed countries has been reported due to global warming and environmental degradation (2–4).

In the Republic of Korea, before 1970s, malaria was epidemic mainly in the urban and rural areas and the country was declared malaria free in 1979 because of the government’s malaria elimination program (5). However*, P. vivax* malaria has recently become endemic since the infection of a soldier stationed in the Demilitarized Zone (DMZ), Paju City in 1993. After an additional incidence of malaria in the areas adjacent to the DMZ in 1994, the annual malaria cases are on a rapid increase and expanding to thousands of people across the country (2, 6). The government remains committed to implementing aggressive malaria approaches by intense management on high-risk geographic regions and enforcing malaria eradication. Meanwhile, more than 1000 cases are still reported annually due to the difficulties in managing areas near the Democratic People’s Republic of Korea (DPRK).

After the re-emergence, about 70% of domestic malaria cases have been reported in the DMZ and in areas adjacent to the DMZ (7, 8). Despite preventive therapies for malaria administered to military personnel stationed on DMZ during every Jun to Oct, malaria is consistently occurring among soldiers serving in the DMZ area (7, 8). The current occurrence of malaria among soldiers near DMZ might be the result of infected mosquitoes originating from the DPRK (4, 9).

The majority of malaria patients are soldiers. From 2005 to 2011, among the total malarial patients except for foreign inflow, almost more than 40% of domestic infections occurred during the military service at ranks of; military personnel (22%), veterans (21%), and civilians (57%) (10). Malarial patients inside troops are treated and managed by military hospitals, but prevention practice behaviors such as malaria chemoprophylaxis for preventive treatment, mosquito control, or mosquito repellent treatments are reported as not being conducted properly (11). Malaria is a mosquito-borne disease and can be prevented using blocking methods for protection from mosquito bites and chemoprophylaxis effective against *P. falciparum*.

Taking malaria drugs in low-risk malaria areas are considered to have greater disadvantages such as side effects or costs than advantages and only the blocking methods are recommended. To avoid mosquito bites, clothes covering arms and legs should be worn or mosquito repellent lotion or cream must be used. In addition, mosquito bed nets are recommended. Limited use of preventive antimalarial drugs can be recommended when the blocking methods are insufficient; however, many obstacles arise for various reasons when travelers try to get prescription and administration (11). Currently, malaria prevention in Korean military troops mainly depends on distributing antimalarial drugs, while education about malaria prevention is not properly conducted. Definitely, providing preventive antimalarial medicines might be an important project to avert malaria, but educating overall knowledge of malaria and encouraging the perception about malaria risks must be preceded by proper use of the drugs in urgent situations. Knowledge and preventive behavior of malaria were assessed only once by the Korea Center for Disease Control and Prevention, and the study were about travelers traveling to malaria-infected regions (5). Studies on soldiers considered, as malaria high-risk group hardly exists.

This study aimed to evaluate the knowledge and practice on malaria among military personnel in nearby DMZ in Paju City and Yeoncheon County, which are malaria high-risk areas, and to provide insights on effective malaria prevention management. The assessment on status concerning the knowledge of malaria and preventive behaviors and on influencing factors can be used as a practical basic data for developing malaria preventive programs.

## Materials and Methods

### Subjects and Procedures

In this self-administered cross-sectional study, the subjects were military personnel and occupation soldiers stationed in nearby DMZ in Paju City and Yeoncheon County. Overall, 300 copies were collected from Paju and Yeoncheon, 150 copies respectively, with the cooperation of the military officials. Typically, 294 copies among collected 300 copies were used in the final analysis and six copies the contents of which were insincere and inconsistent were excluded.

After obtaining the written consent of the subjects, the data were collected over eight days from Feb 3, 2016, to Feb 10, 2016. IRB committee of Sahmyook University (SYUIRB2015-113) approved the ethical considerations of this research study.

### Research Instrument

In Korea, as there are few precedent studies on knowledge and practice of malaria, the questionnaire was made with reference to precedent researchers on infectious diseases (12, 13). Three experts were consulted for the questionnaire and it was verified for the validity of its contents. The questionnaire comprised of 9 questions on general characteristics, 5 on experience and necessity of malaria education program, 11 on knowledge of malaria, and 3 on malaria prevention practice. The question on education experience of malaria was only limited to the period after joining the army service. The questionnaire about knowledge on malaria consisted of 11 questions based on infection routes, symptoms, treatments, and preventive practice on malaria (12). The scores ranged from 0 to 11 and were added together from one point for ‘correct answer’ and zero points for ‘incorrect answer/ignorance’ on each question. The Kuder-Richardson coefficient is an index of the internal consistency reliability and 0.73 (KR=0.73) indicates an acceptable value.

The category of preventive practice behavior consisted of three questions: one question on the use of antimalarial drugs (primaquine, chloroquine) and two questions on the use of repellent lotion/spray or blockage like mosquito nets. Each question was assessed by a 5-point Likert scale ranging from ‘strongly disagree’ on one end to ‘strongly agree’ on the other. The higher the score, the higher the practice of preventing activity, and the value of Cronbach’s α was 0.72, which means an acceptable level of internal consistency.

### Data analysis

Collected data were analyzed after recording and data cleaning using program SPSS ver. 21.0 (Chicago, IL, USA). Knowledge and preventive practice behavior on malaria were analyzed using multiple regression analysis. Statistical significance was proven by significance level (α) of 0.05, and hypothesis in the multiple regression models was assessed using residual analysis and multicollinearity before multiple regression analysis.

## Results

### General characteristics of the subjects

General characteristics of 294 participants were categorized into gender, age, level of education, ranks, and duty. The largest portion of survey respondents was 21 yr old and the smallest portion was above 23 yr of age. The number of people in each age categories of under 20 yr old, 21 yr old, 22 yr old, and above 23 yr old, was 84 (28.6%), 104 (35.4%), 64 (21.8%), and 42 (14.3%), respectively. According to the level of education, the number of people was 60 (20.4%), 92 (31.3%), and 142 (48.3%), in the category of high school, 2∼3 year college attendants, and University attendants or higher education, respectively. Concerning ranks, it was highly distributed on the category of private first class and corporal: 35 privates (11.9%), 104 private first classes (35.4%), 114 corporals (38.8%), and 41 sergeants or higher-ranking soldiers (13.9%). Based on the nature of duty, 169 participants were infantrymen (57.5%) and other 125 respondents (42.5%) varied depending on their duties as driver, gunner, and signalman and so on (Table 1).

**Table 1:** General characteristics (n=294)

**Variable**	**n**	**%**
**Age (yr)**		
Under 20 yr	84	28.6
21 yr	104	35.4
22 yr	64	21.8
Over 23 yr	42	14.3
**Education**		
High school	60	20.4
2-year college attendants	92	31.3
University attendants	142	48.3
**Ranks**		
Private	35	11.9
Private first class	104	35.4
Corporal (3rd class)	114	38.8
Sergeant	41	13.9
**Duty**		
Infantryman	169	57.5
Others (driver, signalman, etc)	125	42.5

### Malaria education experience

Forty-seven people (45.2%) of the 21-yr-old age group had malaria education experience, which is the lowest percentage in age category with statistical significance (*P*<.05). Overall, 64 people (69.6%) of 2∼3-yr of college attendance had malaria education experience, while the lowest percentage of malaria education experience was observed in university attendance or higher education group counting to 71 people (50.0%), which implies statistically significant difference (*P*<.05). With regards to ranking, positive answers to education experience were found mostly among sergeants or higher ranking in 32 people (78.0%) while the least positive answers were noted in 7 private people (20.0%) with statistically significant difference (*P*<.05).

According to the category of duty, soldiers other than infantrymen included more people with education experience counting to 75 people (60.0%) than infantrymen and the difference was statistically insignificant (Table 1). Almost half of the total subjects (147) people, possessed malaria education experience after joining the army service. Many soldiers (56 people (38.1%)) received education on the training place before arrangement of troops, and 35 soldiers (23.8%) received education after six months of arrangement of troops. After joining the army service, 83 soldiers (58.5%) had 2∼4 times of malaria education experience, while 47 soldiers (33.1%) received the education once, and 12 soldiers (8.5%) were educated 5 times or more (Table 2).

**Table 2:** Education experience of malaria (n=294)

**Variable**	**n (%)**
Malaria education experience after joining the army service	147 (50.0)
Malaria education training time after joining the army service	
Training place before the arrangement of troops	56 (38.1)
Within one month after arrangement of troops	28 (19.0)
Within three months after arrangement of troops	28 (19.0)
After six months after arrangement of troops	35 (23.8)
The number of malaria education experience after joining the army service	
1 time	47 (33.1)
2∼4 times	83 (58.5)
5 times or more	12 (8.5)

### Knowledge and behavior regarding practice on malaria prevention

The highest percentage of correct answer questions about knowledge on the item “Being infected with malaria causes you fever” was 83.3%, the second highest percentage was 75.9% for the item “Malaria can be infected through blood transfusion”, and the third highest was 72.4% on “Healthy people are not infected with malaria”. The lowest percentage of correct answer was 19.0% on the item “Only being bitten by a mosquito can lead to development of malaria”, the second lowest was 29.3% on “I know how to take malaria prevention drugs”, and the third lowest was 39.1% on “Malaria may be infected without being bitten by mosquitoes” (Table 3). Calculated by scaling 11 items of knowledge, the mean of knowledge was 5.73 points out of 11 points, approximately half of them answered correctly, which implies low level of knowledge. Educated group with higher level of education (*P*<.01) and higher ranking (*P*<.01) had significantly higher knowledge score than uneducated group (*P*<.001) (Table 4).

**Table 3:** Knowledge and practices on malaria (n=294)

**Items**	**n (%)**
Knowledge (Correct answer rate)	
Malaria may be infected without being bitten by mosquitoes (True).	115 (39.1)
Healthy people are not infected with malaria (False).	213 (72.4)
Person only being bitten by a mosquito can be infected with malaria (False).	56 (19.0)
I know primaquine and chloroquine as preventive drugs (True).	125 (42.5)
If you take malaria prevention drugs, you will not be infected with malaria (False).	155 (52.7)
I know how to take malaria prevention drugs (True).	86 (29.3)
Once infected with malaria, flu-like symptoms appear (True).	192 (65.3)
Eating with malaria patients can cause malaria infection (False).	137 (46.6)
Malaria can be transmitted through blood transfusion (True).	223 (75.9)
Being infected with malaria causes death (False).	139 (47.3)
Being infected with malaria causes fever (True).	245 (83.3)
Preventive practice behavior (rate of agree percentage)	
Use repellent lotion/spray	85 (28.9)
Use mosquito nets	34 (11.6)
Use drugs like primaquine and chloroquine	145 (49.3)

**Table 4:** Knowledge and prevention practice by education and ranks

	**Knowledge**			**Prevention Practice**		
	**M**	**SD**	**t or F**	**M**	**SD**	**t or F**
			(p)			(p)
**Education**			5.61 (.004)			.33 (.721)
High school	5.27	2.97		8.78	3.15	
2-year college attendants	5.23	2.63		8.75	2.78	
University attendants or higher education	6.26	2.41		8.51	2.31	
**Ranks**						
Private	4.91	2.50	4.04 (.008)	7.40	2.52	6.48 (.000)
Private first class	5.33	2.82		8.25	2.95	
Corporal (3rd class)	6.05	2.55		9.02	2.42	
Sergeant	6.59	2.24		9.66	1.89	
**Malaria education experience**						
No	5.20	2.72	t=−3.58 (.000)	7.83	2.60	t=−5.65 (.000)
Yes	6.28	2.45		9.48	2.42	
Total	5.73	2.64		8.64	2.64	
Note: Range: knowledge (0–11), prevention practice (3–15)						

In the category of preventive practice behavior, on the item “Take primaquine and chloroquine appropriately to prevent malaria infection”, 49.3% answered ‘yes’, which means that the medicines are used by half of the soldiers. Next, on the item “Use repellents appropriately” and “Be sure to use mosquito nets while on duty”, 28.9% and only 11.6% answered ‘yes’, respectively (Table 3). The level of preventive practice behavior was low as well as the level of knowledge, as the scaled score using above three items of preventive practice behavior was found to be 8.64 (±2.64) out of 15 possible points. Preventive practice behavior was not related to the level of education. However, significant differences were found in the relation between preventive practice and rank with levels ranging from 9.66 in sergeant, 9.02 in corporal, 8.25 in private first class, and 7.40 in private. The results signify more preventive practice in higher rank (*P*<.001), and that educated group (9.48 points) do more preventive practice than uneducated group (7.83 points) (*P*<.001) (Table 4).

### Factors affecting malaria knowledge and prevention practice

To figure out the factors influencing malaria knowledge and prevention practice, general characteristics of the subjects (ranks, level of education, and education experience), with significant results in univariate analysis were used as independent variables. For finding out factors affecting knowledge on malaria (dependent variable), general characteristics were used as independent variables, and for preventive practice behavior (dependent variable), both general characteristics and knowledge on malaria were used as independent variables in multiple regression analysis.

Using multiple regression analysis, the factors of independent variables affecting knowledge on malaria were assessed. The factors influencing the knowledge are the level of education (β=.24, *P*<.001), malaria education experience (β=.21, *P*<.001), rank 1 (β=.13, *P*<.05), and rank 2 (β=.13, *P*<.05) in order of importance (F=9.62, *R*^2^=.12, *P*<.001). In other words, knowledge on malaria was found to be significantly higher in the group of university attendants or higher education, experienced on malaria education, and sergeant or corporal as compared to the group of 2∼3 yr college attendance or lower education, inexperienced on malaria education, and private or private first class, respectively. The adjusted coefficient of determination (R^2^) was 0.12, and the variable explainability emerged as 12% of the total variables (Table 5).

**Table 5:** Factors affecting malaria knowledge and prevention practice

	**Malaria knowledge**				**Malaria prevention practice**			
	**β**	**SE**	**t**	**P**	**β**	**SE**	**t**	**P**
Rank 1	.13	.46	2.12*	.035	.11	.45	1.85	.065
Rank 2	.13	.32	2.14*	.033	.11	.31	1.82	.070
Level of education	.24	.30	4.20***	.000	−.05	.30	−.88	.380
Experience of malaria education	.21	.31	3.58***	.000	.22	.30	3.89***	.000
Malaria knowledge					.25	.06	4.37***	.000
F(p)	F=9.62 (p=.000)				F=12.45 (p=.000)			
R^2^	R^2^=.12				R^2^=.18			
Note: Rank 1(0=others, 1=sergeant or more), Rank 2(0=others, 1=corporal)								
Level of education (0=High school, two year college, 1=University or more)								

Because of multiple regression analysis, knowledge (β=.25, *P*<.001) and malaria education experience (β=.22, *P*<.001) were observed as significant factors affecting the practice (F=12.45, *R*^2^=.18, *P*<.001). Rank 1 (β=.11, *P*<.1) and rank 2 (β=.11, *P*<.1) were marginally significant (*P*<.1) because the factors were not significant at the level of 0.05 but were significant at the level of 0.1. Therefore, knowledge on malaria and education experience was proportional to the score of preventive practice (<.001). In addition, sergeants and corporals scored higher points than private or private first class on the level of significance 0.1 (*P*<.1). The variable explainability turned out to stand as 18% of the total variables, as the adjusted coefficient of determination (R^2^) was 0.18 (Table 5).

## Discussion

The Republic of Korea was declared malaria free in 1979 as a result of the government’s malaria elimination program, but since the incidence related to a soldier stationed in Demilitarized Zone (DMZ), Paju City in 1993, thousands of annual incidences have been reported (5). Especially, military personnel or veterans are important subjects to be considered for preventive practice behavior because they occupy more than 40% of domestic infection patients among the total malarial patients except for the foreign inflow (10). Recent climatic changes in Korea towards sub-tropical climate might lead to more incidences of malaria; henceforth, development of strategies for prevention of disease is of immense importance. Prevention is the first priority as there are no commercially available malaria vaccines now. Considering the present circumstances, the aim of this study was to provide basic data required for developing malaria prevention program by carrying out research on knowledge related to malaria and preventive practice amongst soldiers stationed at DMZ, who are at high risk of developing malaria. Gaining better understanding of knowledge about infectious disease and prevention practice are important basis for developing preventive program for infectious diseases (14). Domestic researches on malaria are mainly concentrating on epidemiological characteristics, and few types of research are dealing with knowledge and practice amongst Korean people. Most of the Koreans consider malaria as a foreign inflow infectious disease and not endemic in Korea.

Among the questions on the category of malaria knowledge level, symptoms and infection routes, the subjects were well educated but the knowledge level related to preventive practice on malaria was low. In other words, the fact that infected mosquitoes transmit malaria and one of its symptoms are high fever was well known; however, the subjects were not educated enough how to prevent malaria. The mean of knowledge was 5.73 out of possible 11 points, which shows the low level of knowledge as the percentage of correct answer was just half of the total questions. Knowledge on malaria concerning mosquitoes and high fever is widespread in foreign countries including Korea (13). The knowledge scores are affected by the level of education, ranks, and education experience on malaria. In addition, knowledge on malaria is affected by the similar above three factors: the level of education, ranks, and education experience on malaria. Knowledge on malaria is significantly higher in the group of university attendants or higher education, experienced on malaria education, and sergeant or corporal as compared to the group with 2∼3 yr of college attendance or lower education, inexperienced on malaria education, and private or private first class, respectively. As stated by the report of the Korea Center for Disease Control and Prevention (2008), the level of education is a significant variable affecting health conditions or disease. Besides, the uneducated and elementary school graduates had 9.8 times and 4.3 times higher infection risks, respectively, than university graduates (15). Knowledge on malaria is proportional to socioeconomic status and the level of education (12).

Almost half of the respondents stated that they take primaquine or chloroquine in an appropriate manner to prevent malaria infection; however, the preventive behavior practice rates were low, because only 28.9% and 11.6% of respondents had used repellents and mosquito nets at the work place, respectively. Knowledge level and education experience on malaria were significant influencing factors on preventive practice. There existed differences in protecting oneself from malaria infection among residents of city, construction sites, village, and slums (12). City respondents were more likely to use liquid repellents and mosquito sprays, perhaps due to their ease of use and higher incomes in the city, and respondents from construction sites were more likely to use bed nets because local government distributes free bed nets in those areas. The preventive practice differed based on incomes of residents in city, village, and slums. Environmental factors were not considered in this study because the subjects of this study were soldiers. Proper knowledge on health and education experience was observed as incredibly important factors in this study.

Thus, periodic education and guide materials are the first priority to alert soldiers and civilians living in the infected areas to prevent malaria. The present study has certain limitations; generalizing the result of the present study is restricted because the subjects were limited to military personnel stationed in a specific area. However, the present study seems to be very meaningful because subjects were the highest risk group for malaria in Korea and there exist no precedent research on the knowledge on malaria and practice with reference to such population. In the near future, policy and education system for preventing malaria and the quality of life of the soldiers can be improved.

## Conclusion

About 20 yr have passed since the re-emergence of malaria in Korea. The government aims to eradicate malaria by decreasing the incidence rate yearly, but eradication is not an easy task. In low-risk areas of malaria like Korea, instead of taking antimalarial drugs, wearing clothes with long sleeves or using mosquito repellent lotions or creams can be a proper way to avoid mosquito bites. Above all, promoting awareness and providing education should be executed to emphasize the importance of preventive practice. It seems necessary to educate military personnel about preventive measures against malaria as the transmission route of malaria is clearly identified.

## Ethical considerations

Ethical issues (Including plagiarism, informed consent, misconduct, data fabrication and/or falsification, double publication and/or submission, redundancy, etc.) have been completely observed by the authors.

## References

[1] WHO (2014). World malaria report 2014. http://www.who.int/malaria/publications/world_malaria_report_2014/en/

[2] Kho WG (2007). Reemergence of Malaria in Korea. J Korean Med Assoc, 50 (11): 959–66.

[3] Lee KS Kim TH Kim ES (2009). Chloroquine-resistant Plasmodium vivax in the Republic of Korea. Am J Trop Med Hyg, 80 (2): 215–7. 19190216

[4] Yeom JS Park JW (2008). Status of vivax malaria after re-emergence in South Korea. Infect Chemother, 40 (4):191–8.

[5] KCDC (2014). Epidemiological investigation of infectious diseases in Korea annual report 2013. Korea Center for Disease Control.

[6] Chai I Lim G Yoon S Oh W Kim S Chai J (1994). Occurrence of tertian malaria in a male patient who has never been abroad. Korean J Parasitol, 32 (3): 195–200. 7953245 10.3347/kjp.1994.32.3.195

[7] Park JW Kim YA Yeom JS Yoo JS Yang BG Chai JY (2001). Status of Vivax Malaria in the Republic of Korea in 2000. Korean J Infect Dis, 33 (4): 280–4.

[8] Chai JY (1997). Reemergence of Malaria in Korea. J Korean Med Assoc, 40 (6): 728–33.

[9] Yeom J-S Kim T-S Oh S (2007). *Plasmodium vivax* malaria in the Republic of Korea during 2004–2005: changing patterns of infection. Am J Trop Med Hyg, 76 (5): 865–8. 17488906

[10] KCDC (2012). AFMRI. Military malaria seminar.

[11] Park JW Hong JY Yeom JS Cho SR Oh DK (2009). Evaluation of the Current Status of Malaria Elimination Project in the Republic of Korea and Suggestion for Improvement of its Efficacy. Infect Chemother, 41 (1): 42–53.

[12] Dhawan G Joseph N Pekow PS Rogers CA Poudel KC Bulzacchelli MT (2014). Malaria-related knowledge and prevention practices in four neighbourhoods in and around Mumbai, India: a cross-sectional study. Malar J, 13: 303. 25102949 10.1186/1475-2875-13-303 PMC4248467

[13] Hlongwana KW Mabaso ML Kunene S Govender D Maharaj R (2009). Community knowledge, attitudes and practices (KAP) on malaria in Swaziland: a country earmarked for malaria elimination. Malar J, 8:29. 19228387 10.1186/1475-2875-8-29 PMC2649151

[14] Sohn A Park S (2012). HIV/AIDS knowledge, stigmatizing attitudes, and related behaviors and factors that affect stigmatizing attitudes against HIV/AIDS among Korean adolescents. Osong Public Health Res Perspect, 3 (1): 24–30. 24159483 10.1016/j.phrp.2012.01.004 PMC3738688

[15] KCDC (2008). Risk factors of malaria in Ganghwa. Korea Center for Disease Control.

